# Elements of episodic memory: lessons from 40 years of research

**DOI:** 10.1098/rstb.2023.0395

**Published:** 2024-09-16

**Authors:** Gema Martin-Ordas, Alexander Easton

**Affiliations:** ^1^ Division of Psychology, University of Stirling, Stirling, UK; ^2^ Department of Psychology, Durham University, Durham, UK

**Keywords:** Episodic memory, Autonoetic awareness, Mental Time Travel

## Abstract

40 years ago, Endel Tulving published his hugely influential *Elements of Episodic Memory* (Oxford: Clarendon Press, 1983). For the first time, this discussed the details of episodic memory (i.e. the ability to remember personal past events), including a specific conscious experience. Ten years later, Tulving defined the ability to mentally project oneself in time to be the critical feature distinguishing episodic from semantic memory (‘What is episodic memory?’ *Curr*. *Dir*. *Psychol*. *Sci*. **2**, 67–70, doi:10.1111/1467-8721.ep10770899). In this conception, the conscious experience of episodic memory captures the experience of reliving a personal event as it was experienced in the past, while the same ability allows a potential symmetry between remembering the past, and our ability to project into an imagined future. With the recent passing of Endel Tulving, this theme issue offers an opportunity to question our understanding of mental time travel in full.

This article is part of the theme issue ‘Elements of episodic memory: lessons from 40 years of research’.

## 1. Overview of the theme issue

The conception of mental time travel (MTT) as originally outlined allowed such a conscious experience to be a unique feature of human memory [1–3]. In the years since these original positions were posited, this has provided a challenge for researchers across multiple fields to test and challenge this conjecture. The editors have collated a theme issue that reflects the nature of these arguments over the last 30 or more years, reflecting critical questions those early positions raised. At what point (and how) does MTT develop? Is MTT unique to humans? What cognitive processes underlie episodic memory and future thinking? How does the concept of MTT extend to artificial memories, other forms of projection?

Our issue follows a clear thematic structure, and each contribution adds to, and builds on, previous contributions to offer a genuinely novel and timely perspective on the topic. The main themes of this theme issue are as follows.

### (a) Historical perspective of the concept of MTT

To begin any discussion of MTT and the questions that face us today, we need to first consider it in both historical and conceptual terms. Hoerl and McCormack outline the historical perspective on the evolution of Tulving’s ideas about episodic memory, as well as the challenges that remain around characterizing episodic memory and, in particular, the elements related to MTT (for more information about Tulving’s life and work please see [4]). Davies and Clayton review the different methodologies used to assess episodic (like) memory in animals and argue that a multidimensional approach is required to understand the evolution of this ability, challenging Tulving’s assumptions about episodic memory being uniquely human. Latham, Miller and Pedersen’s contribution then develops a novel theoretical framework from which to discuss and consider the different types of temporal representations that are required for MTT.

### (b) The development of MTT

To understand MTT, it is important to explore its development in humans. Is such an ability innate, and if not when and how does it develop across the lifespan? Ayson and Atance first propose a functional and naturalistic methodological approach to the study of future thinking in children and show that children by the age of 3 already demonstrate sophisticated forms of future thinking. Coughlin and colleagues take a different approach to this question and borrow methodology previously used with animals to assess the development of episodic memory. The results indicate that integration of semantic knowledge and decision-making is critical for succeeding in what–where–when tasks. Ortiz-Tudela and collaborators then examine the relation between schema-driven predictions and episodic memory across lifespan. Their findings support to the existence of two separate mechanisms underlying episodic memory encoding and highlight how these different mechanisms come into play at various stages of life.

### (c) The evolution of MTT

The original conception of MTT as uniquely human raises important challenges to understanding whether other animals show the same capability. In their contribution, Osvath and colleagues adopt a phylogenetic perspective that offers a deep insight into the evolution of this ability. They draw a natural history to elucidate how events that occurred hundreds of millions of years ago have been fundamental for the emergence and evolution of MTT. Healy and colleagues further outline the importance of episodic memory in animals through examples from the field in which such memories enable decisions to be made about the animals’ futures. Crystal proposes specific requirements for animal episodic memory and highlights the ability of rats to meet these same requirements. Martin-Ordas extends work on this area by presenting a study with invertebrates, in this case insects, assessing the constructive nature of their memories. This contribution represents an attempt to bring into the comparative arena the study of memory errors—noting that, until now, this research area has mostly focussed on animals’ successful remembering. Finally, Collaro and collaborators present an interdisciplinary perspective on episodic memory and MTT across species, suggesting that the two might be somewhat different, and dissociable, cognitive processes, supporting distinct aspects of behaviour.

### (d) Rethinking MTT

Having understood the development of the concept of MTT, and the challenges to that concept in other species, how might we now rethink the concept to deal with our current ideas of memory? Gentry and Buckner argue that semantic and episodic memory display systematic transitional forms in human psychology—suggesting that attempts to establish a distinction between these transitional forms would just be arbitrary. They offer a continuum view and argue that this view will allow to proceed more fruitfully in fields such as comparative psychology. Addis and Szpunar consider whether MTT is indeed unique to episodic memories and present a new model framework to account for the range of past, present and future representations that can be created by the human mind. Mahr and Schacter propose an updated account of episodic recombination. The authors argue that episodic recombination should be distinguished from the mechanisms determining the temporal orientation of episodic representations—suggesting that MTT is integrated by the episodic representations and their temporal representation. De Brigard brings a controversial proposal in his paper: autonoetic awareness is not necessary for episodic memory. Instead, de Brigard proposes a functional and computational characterization of episodic memory. In this framework, the phenomenological aspect of episodic memory is a contingent feature of the retrieval process and, thus a feature that can be empirically examined. Cheng then presents a framework in which episodic memories rest on traces of the episode, which are then built on and enriched by semantic information to build a scenario for the past experience.

### (e) New perspectives on MTT

As well as better understanding the conceptualization of episodic memory, and MTT in particular, how might we adapt this knowledge to generate new perspectives and insight? Redshaw provides a framework in which the relation between recursion and MTT is examined. Redshaw’s proposal offers novel ways to empirically investigate and assess the development and evolution of MTT and its relationship with other cognitive abilities. Andonovski and colleagues question whether the recent developments in our understanding of remembering and imagining might even challenge the concept of episodic memory itself. Dafni-Merom and collaborators extend the concept of MTT by presenting an fMRI study of peoples’ projection into political and moral, as well as temporal, futures. They propose that MTT should be considered ‘mental–experiential travel’ and that while self-reference might be related primarily to time, self-projection is a domain-general construct. Prescott and Dominey build on work in the development of episodic memory and apply it to cognitive architectures in robotics. In particular, cognitive architectures that include a language capacity show progress towards the construction of a narrative self and the capacity for autobiographical memory in robots. Finally, Boyle and Blomkvist point to the nature of AI systems that have been inspired by episodic memory, and how the ways in which they are similar to, and differ from, human behaviour can offer a role in directing future episodic memory research by highlighting hypotheses worthy of further pursuit.

## 2. Conclusion

MTT perhaps allows us today more understanding of the nature of memory than Tulving ever envisioned. The challenge to address MTT in animals other than humans has not only offered us new insights into the nature of animal memory but also raised questions about the nature of the conscious experience in these animals. Exploring the way in which MTT develops in children offers unique insights again into the nature of memory and complex understanding in development. As a concept, MTT has also allowed us to reconceptualize other elements of human (and artificial) intelligent behaviours, and they, in turn, offer us a chance to further reflect on MTT and episodic memory.

Perhaps most importantly, this theme issue highlights the value of an interdisciplinary perspective on MTT and associated memory. Too often discussions around MTT develop within several fields independently of one another. The need for discussion across these disciplinary boundaries is obvious in this issue where different disciplinary perspectives offer challenges to others and where a broader perspective will allow better integration of our models of memory, and the application of those models to the real world.

The editors of this theme issue have long worked on an interdisciplinary approach to episodic memory and MTT, and are passionate about the value such approaches offer. Martin-Ordas’s work on MTT has combined cognitive, developmental and comparative psychology, and has recently incorporated a philosophical view in order to address the role of time in MTT. Easton’s work has brought together psychologists, neuroscientists, anthropologists and philosophers, as well as scholars across the humanities. The nature of this theme issue highlights the value that can be gained through a genuinely interdisciplinary perspective, where novel insights can be developed to challenge, test and generate important ideas about cognitive function.

## Data Availability

This article has no additional data.
